# Endoplasmic reticulum stress in the pathogenesis of early-onset pre-eclampsia

**DOI:** 10.1016/j.preghy.2010.12.002

**Published:** 2011-01

**Authors:** Graham J. Burton, Hong-Wa Yung

**Affiliations:** Centre for Trophoblast Research, Department of Physiology, Development and Neuroscience, University of Cambridge, Cambridge CB2 3EG, UK

**Keywords:** Endoplasmic reticulum, Placenta, Pre-eclampsia, Protein synthesis

## Abstract

Recent data have provided molecular evidence of high levels of endoplasmic reticulum stress in non-laboured placentas from cases of early-onset pre-eclampsia. Endoplasmic reticulum stress is intricately linked to oxidative stress, and the two often share the same aetiology. In the case of pre-eclampsia this is likely to be placental malperfusion, secondary to deficient conversion of the spiral arteries. Endoplasmic reticulum stress activates a number of signalling pathways aimed at restoring homeostasis, but if these attempts fail then the apoptotic machinery may be activated. The potential consequences for placental development and function are numerous and diverse. Inhibition of protein synthesis results in lower levels of many kinases, growth factors and regulatory proteins involved in cell cycle control, and experiments *in vitro* reveal that endoplasmic reticulum stress slows cell proliferation. Chronic, low levels of stress during the second and third trimesters may therefore result in a growth restricted phenotype. Higher levels of endoplasmic reticulum stress lead to activation of pro-inflammatory pathways, a feature of pre-eclampsia that may contribute to maternal endothelial cell activation. These findings emphasise the complexity of cellular responses to stress, and the need to approach these in a holistic fashion when considering therapeutic interventions.

## 1 Introduction

Placental dysfunction has long been implicated in the pathophysiology of pre-eclampsia. The evidence is particularly compelling in the early-onset form of the syndrome, to the point that this has been referred to as ‘placental pre-eclampsia’ [1]. In the classic two-stage model of the syndrome, deficient spiral arterial conversion is thought to lead to placental oxidative stress through malperfusion, which induces the placenta to release factors into the maternal circulation that cause endothelial cell activation [2,3]. There is a wealth of data indicating that placental oxidative stress occurs in the early-onset form of the syndrome [4,5], and experiments conducted on term villous explants *in vitro* have confirmed that oxidative stress is a sufficient stimulus for the release of an array of cytokines and pro-inflammatory factors from the trophoblast [6]. The explant model system has enabled the intermediary signalling pathways activated to be identified [7], and the relevance of these to the *in vivo* situation is confirmed by the fact that the same changes are seen following labour, when placental oxidative stress is induced through ischaemia–reperfusion secondary to uterine contractions [8]. Oxidative stress can cause widespread disruption of cell function however, and rarely occurs in isolation to other cell stress responses. Over the last decade, close links have been identified between oxidative stress and endoplasmic reticulum (ER) stress, with each being able to induce the other [9–11].

The ER is most commonly recognised for its role in the post-translational modification of proteins, but recently it has emerged that the organelle is also a central co-ordinator of diverse signalling pathways regulating cell metabolism, proliferation and death. This role is perhaps not surprising given that protein synthesis is central to cellular integrity and function, and is a heavily energy dependent process requiring an adequate supply of nutrients and oxygen. Disturbances of ER function lead to a state known as ER stress, and activate a series of evolutionarily conserved signalling pathways collectively referred to as the Unfolded Protein Response (UPR). Initially, the UPR aims to restore ER homeostasis, but if these attempts fail then the apoptotic cascade is activated. These pathways are now recognised as playing a central role in the pathophysiology of chronic diseases, such as neurodegenerative diseases and diabetes [12]. Here, we consider evidence that they also contribute to the placental pathology in cases of early-onset pre-eclampsia.

## 2 The endoplasmic reticulum

The ER consists of a series of interconnecting flattened membranous sacs with an intraluminal space of 20–30 nm located in the perinuclear region of a cell, being continuous with the outer membrane of the nucleus. It is responsible for the synthesis and post-translational folding and assembly of all secretory and membrane-bound proteins, including hormones, growth factors and receptors. The amount of ER within a cell therefore differs according to the cell’s function, being large in cells with a high secretory activity, such as beta pancreatic cells and the syncytiotrophoblast. Proteins destined for the ER are identified by a short leading sequence of hydrophobic amino acids at the N-terminus end, which is recognised by the signal recognition particle, a ribonucleoprotein within the cytosol. Synthesis of all proteins starts on a ribosome free within the cytosol, but when the ER signal sequence is recognised by the signal recognition particle the latter binds the ribosome complex to a receptor on the outer surface of the ER membrane. This arrangement creates the characteristic beaded appearance at the ultrastructural level referred to as rough endoplasmic reticulum, and enables the nascent polypeptide chain to be threaded through a translocation channel, the translocon, into the ER lumen. Once within the lumen, the signal sequence is cleaved, and chaperone proteins bind to the polypeptide chain to prevent premature and inappropriate folding. Glucose-regulated protein GRP78/BiP, a member of the HSP70 family, binds to hydrophobic amino acid groups of secretory proteins, and facilitates folding through the hydrolysis of ATP by an ATPase domain. Calnexin and calreticulin are specifically involved in the folding of glycoproteins, binding to monoglucosylated N-linked glycans [13].

The ER also acts as a major intracellular store of calcium, and the concentration within the lumen is often several thousand-fold higher than in the cytosol, reaching millimolar levels [14]. This gradient is maintained by the activity of Ca^2+^-ATPases within the ER membrane, and is considered necessary for functioning of the protein folding machinery and chaperone proteins [15].

## 3 Protein folding and assembly

Correct folding into the secondary and tertiary conformation, and assembly into multimeric complexes, is essential for the functional competence of many proteins. For the extracellular proteins passing through the ER this most commonly involves the formation of covalent disulfide bonds between cysteine side chains, either within different parts of a polypeptide chain or between two such chains. For example, the alpha sub-unit of human chorionic gonadotropin contains five disulphide bonds, while the beta sub-unit contains six [16]. Formation of disulfide bonds is an oxidative event, and consequently the ER is a site of significant production of reactive oxygen species (ROS) within the cell [17]. During the formation of a disulfide bond electrons are first removed from the cysteine thiol groups by the enzyme protein disulfide isomerase, PDI, and are transferred to molecular oxygen by the enzyme ER oxidoreduction, ERO1, using FAD as an intermediate. Because of the kinetics, full reduction of oxygen may not occur, in which case ROS intermediates such as hydrogen peroxide will be produced [17]. Consequently, the ratio of reduced to oxidised glutathione, the principal redox buffer within the ER lumen, is approximately 3:1 compared to that of approximately 100:1 in the cytosol [18].

If the protein load exceeds the capacity of the folding machinery then unfolded or misfolded proteins will accumulate within the ER lumen. This is a potentially dangerous situation for a cell as it may lead to loss of function of membrane receptor proteins or secreted hormones. Equally, considerable energy may be spent attempting to refold the proteins, resulting in depletion of reserves and excessive generation of ROS. In order to guard against these eventualities, cells have evolved the UPR [19,20]. This aims to restore homeostasis within the ER lumen by; (i) reducing the burden on the folding machinery through limiting the number of new polypeptide chains entering the ER lumen, (ii) increasing the capacity of the machinery by synthesising more ER, (iii) generating more chaperone proteins, and (iv) removing accumulated misfolded proteins through stimulation of the ER-associated proteosomal degradation pathway (ERAD). If homeostasis cannot be re-established then the apoptotic cascade is activated so that the cell is removed in a co-ordinated manner.

## 4 The unfolded protein response

The UPR comprises three conserved signalling cascades. The sensors are ER transmembrane proteins, each of which has a luminal domain projecting into the lumen and a cytoplasmic domain that transmits the signal downstream. Under normal conditions the sensors are held in an inactive state by the binding of GRP78, and activation occurs when this is titrated away by competitive binding to accumulated proteins within the lumen.

PERK (double-stranded RNA-dependent protein kinase (PKR)-like ER kinase), is a Ser/Thr protein kinase. Upon release from GRP78 it dimerises and undergoes autophosphorylation, activating the kinase domain. The principal target of p-PERK is eukaryotic translation-initiation factor 2α (eIF2α), a sub-unit of the eIF2 complex that mediates binding of tRNAs to the ribosomal sub-unit. Phosphorylation of eIF2α inhibits its activity, therefore rapidly blocking further entry of nascent proteins into the ER lumen and reducing the protein load within the lumen. Paradoxically, although there is a global reduction in protein synthesis, the translation of selected mRNAs is favoured under these conditions. mRNAs containing either small upstream open reading frames or internal ribosome entry sites are able to by-pass this block, and so an increase in their encoded proteins is observed. A key example is activating transcription factor-4, ATF4, which translocates to the nucleus and activates *GADD34* (growth-arrest DNA damage gene 34) and *CHOP*, amongst others. GADD34 provides a negative feedback on protein synthesis inhibition by dephosphorylating p-eIF2α, allowing translation to resume if ER homeostasis has been restored.

ATF6 (activating transcription factor 6) translocates to the Golgi following release from GRP78, where it is cleaved into an active form that migrates to the nucleus and regulates transcription of *GRP78*, *CHOP* and *Xbp1*. In this way synthesis of the ER chaperone proteins is increased under low-grade stimulus, or apoptosis is induced under more severe stress.

Ire1 (inositol-requiring transmembrane linase/endonuclease 1) dimerises after release from GRP78, and contains both an endoribonuclease domain and a Ser/Thr kinase domain. The former splices *Xbp1* mRNA, generating a functional transcription factor that binds to the UPR elements of many genes involved in ER function. It notably up-regulates lipid biosynthesis, forming more ER cisternae, genes involved in the protein folding machinery, and enzymes of the ERAD pathway promoting clearance of misfolded proteins. Importantly, in the context of pre-eclampsia, Ire1 can also activate pro-inflammatory pathways through its kinase domain. Acting through TRAF2 (tumour necrosis factor-receptor-associated factor 2) and ASK1 (apoptosis signal-regulating-kinase 1) it stimulates the p38 MAPK, JNK and NFB pathways, leading to the release of inflammatory cytokines.

If the UPR fails to overcome the accumulation of misfolded proteins, a final signalling pathway is triggered to eliminate the cell by activation of cleavage of caspase 4 (caspase-12 in mouse), located in the ER membrane [21]. This ER-specific caspase is able in turn to activate the downstream effector caspase 9 directly, independent from the Apaf1 and mitochondrial cytochrome *c* pathway [22]. In addition, CHOP induced by PERK and ATF6 can sensitize cells to apoptosis, through suppression of the anti-apoptotic factor B cell lymphoma-2 (Bcl-2) gene expression and upregulation of Bim, a proapoptotic BH3-only member of the Bcl-2 family [23,24].

## 5 Evidence of graded activation of the UPR in trophoblast-like cells

The UPR thus provides an integrated response to the accumulation of unfolded or misfolded proteins within the ER lumen, with synergy and some overlap in function between the signalling pathways. Teleologically, it might be expected that the response would act in a graded fashion, with initial attempts to restore ER homeostasis being followed later by activation of the apoptotic cascade if they fail. Application of increasing concentrations of tunicamycin, a blocker of glycosylation and hence a powerful inducer of ER stress, to JEG-3 choriocarcinoma cells has shown that this is indeed the case [25]. Phosphorylation of eIF2α is seen at the lowest doses, followed by upregulation of the chaperone proteins GRP78 and 94, and splicing of *Xbp1* mRNA as the concentration rises. An increase in CHOP is seen at the higher concentrations of tunicamycin, and is associated with elevated rates of apoptosis. Equally, activation of the different pathways can be separated temporally. Application of a non-lethal dose of tunicamycin to JEG-3 cells results in rapid phosphorylation of eIF2α, and a slower increase in the chaperone proteins. No increase in CHOP is observed with this low-grade stimulus. There is therefore considerable evidence of a graded response from this model system, although how this is regulated at the molecular level is currently unknown.

## 6 Evidence of placental ER stress in pregnancies complicated by early-onset pre-eclampsia

**Fig. 1:**
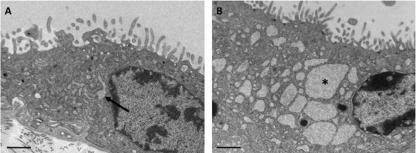
Electron micrographs of the syncytiotrophoblast in (A) a normal placenta, and (B) a case of pre-eclampsia. Both placentas were delivered at term following caesarean section. In the normal placenta the cisternae of endoplasmic reticulum display only minimal dilation (arrow), whereas in the pre-eclamptic placenta they are widely dilated (asterisk) and filled with an amorphous proteinaceous precipitate. Scale bars = 1 μm.

Dilation of the ER cisternae within the syncytiotrophoblast indicative of a loss of ER homeostasis has been previously reported in cases of pre-eclampsia [26], although its physiological significance in terms of the UPR was not appreciated at the time (Fig. 1). Similar dilation has also been associated with anoxia in placental samples that are not fixed immediately after delivery, or are malperfused *in vitro* [27].

We have recently provided the first molecular evidence of activation of the UPR in placentas from cases of normotensive intrauterine growth retardation (IUGR) and from IUGR associated with early-onset pre-eclampsia (IUGR+PE) [25]. In both sets of placentas we observed phosphorylation of eIF2α, which was absent in control placentas delivered at term by caesarean section. The degree of phosphorylation was greater in the IUGR+PE cases, suggesting a higher level of ER stimulation. Commensurate with this hypothesis, we observed increased levels of CHOP in the IUGR+PE cases, but not in IUGR alone, and immunohistochemistry localised this principally to the syncytiotrophoblast and the endothelial cells of the fetal capillaries. There was also a rise in GRP94 in IUGR+PE, but not in IUGR alone. No change in GRP78 was observed in either pathology, and interestingly was also not found under oxygen-glucose deprivation in JEG-3 cells where there was an increase of P-eIF2α and CHOP and cleavage of *Xbp-1 mRNA* [28]. Extensive splicing of *Xbp1* mRNA was seen in both IUGR and IUGR+PE placentas, and was not significantly different between the two conditions.

## 7 Significance of ER stress in the pathophysiology of pre-eclampsia

Given both the morphological and molecular evidence of ER stress in early-onset pre-eclamptic placentas, what might the significance be for the pathogenesis of the disorder?

ER stress can be induced by many stimuli, and the precise cause in pre-eclampsia is not known. However, an ischaemia–reperfusion-type injury is a strong possibility given the associated spiral arterial pathology. Early-onset pre-eclampsia, along with IUGR, has long been associated with deficient conversion of the endometrial spiral arteries secondary to poor trophoblast invasion. Conversion normally extends from the placental interface as far as the inner third of the myometrium, and is associated with the loss of smooth muscle and the elastic lamina from the vessel walls. Exact quantification of the degree of conversion is difficult, given the small size and number of the samples available for study. However, there is general agreement between studies that the myometrial segments of the arteries are more adversely affected in pathological pregnancies than the decidual segments, and that the deficit is greater in cases associated with pre-eclampsia than IUGR alone [29–33]. The portion of the artery just below the endometrial/myometrial boundary represents a specialised highly contractile segment [34], that is thought to prevent excessive blood loss at the time of menstruation. In view of this, we recently proposed that retention of this segment in cases of pre-eclampsia predisposes to spontaneous vasoconstriction, and hence to an ischaemia–reperfusion-type injury [35,36]. Cerebral ischaemia is a powerful inducer of the UPR [37], and subjecting JEG-3 cells to hypoxia-reoxygenation causes phosphorylation of eIF2α [25]. This situation may be made worse by changes in posture, which in the bipedal human can influence uterine blood flow [38], or heightened uterine contractility, as maternal placental blood is reduced during a contraction [39].

The intervening steps *in vivo* are unclear at present, but various possibilities exist. Episodes of ischaemia will deplete intracellular concentrations of glucose, which may restrict normal glycosylation within the ER, activating the UPR. Alternatively, ischaemia will reduce intracellular levels of ATP, compromising the functioning of the GRP chaperone proteins, and possibly also the Ca^2+^-ATPase ionic pumps within the ER membrane. Ischaemia may also have a more direct effect on calcium release from the ER by altering the redox balance within the cell, affecting thiol groups on the calcium channel proteins [40]. Calcium imbalance may further result from competitive binding of GRP78 to misfolded proteins, for under normal conditions GRP78 serves to plug unoccupied translocons, preventing leakage. Loss of calcium from the ER lumen will compound the situation by compromising the protein folding machinery, and by activating calcium dependent signalling pathways within the cytsol. Ultimately, these could lead to opening of the mitochondrial membrane transition pore, with subsequent loss of mitochondrial function and generation of ROS. We have previously demonstrated that hypoxia-reoxygenation of villous explants leads to opening of the pore, and activation of apoptosis within the syncytiotrophoblast [41]. Further work is required to tease apart these various possibilities, but the complex interactions between oxidative and ER stress mean that once one is initiated the other is likely to follow soon after through feed-forward mechanisms.

In many instances pathological activation of the UPR is a one-off event, following for example stroke or myocardial infarction. As mentioned earlier, phosphorylation of eIF2α and inhibition of protein synthesis are usually transient events, for activation of ATF4 leads to upregulation of the phosphatase GADD34. However, the precipitating vascular insult to the placenta in pre-eclampsia is likely to be of a lower grade than that in stroke, and also of a repetitive nature. To mimic this *in vitro* we have exposed JEG-3 cells to repetitive cycles of hypoxia-reoxygenation and observed sustained phosphorylation of eIF2α and activation of the UPR. We predict therefore that the ER stress is of a chronic nature, dating most likely from the time of onset of the maternal circulation at the end of the first trimester.

The consequences for placental function are manifold, and are just beginning to be explored [42]. In the absence of more extensive data, four aspects will be considered here; the impact on placental development, the impact on placental function, the impact on placental apoptosis, and the impact on inflammation.

## 8 Impact on placental development

The protein synthesis inhibition seen as a result of the phosphorylation of eIF2α has a number of consequences for placental development, since a range of kinases and other regulatory proteins are affected. We have observed that levels of all three isoforms of AKT are reduced at the protein, but not at the mRNA level, in IUGR and IUGR+PE placentas, suggesting that translation is suppressed [25]. A reduced level of total AKT is also observed in JEG-3 cells following exposure to hypoxia-reoxygenation, glucose deprivation or tunicamycin, and a pulsed radiolabelled methionine experiment confirmed reduced protein synthesis [28]. AKT plays a central role in regulating cell proliferation, and this loss of activity is likely to have a severe detrimental effect on placental development. Knock-out of *Akt1* in the mouse results in placental and fetal IUGR, and although there may be compensatory increases in Akt2 and Akt3, there is a close linear correlation between the level of phospho-Akt and placental weight [25,43].

Another protein severely affected by the UPR is cyclin D1, and levels have been reported to be severely reduced following ischaemia in the brain [44]. We found cyclin D1 to be depleted in IUGR and IUGR+PE placentas [25].

These two effects on AKT and cyclin D1 are likely to have a major impact on the rate of proliferation of placental cells. This rate is impossible to estimate longitudinally during pregnancy, but counts of cytotrophoblast cells immunopositive for proliferation markers at delivery reveal a lower frequency in IUGR placentas than in controls [45]. Equally, exposure of JEG-3 cells to low-dose tunicamycin or repetitive cycles of hypoxia-reoxygenation slows their proliferation whilst increasing phosphorylation of eIF2α [25]. Although there can be no direct proof that these changes in AKT and cyclin D1 are causal, they are consistent with the smaller placental phenotype observed in IUGR, and to a greater extent in IUGR+PE [46].

In addition, the syncytiotrophoblast secretes a wide array of growth factors, such a vascular endothelial growth factor and members of the insulin-like growth factor family, that may act in an autocrine or paracrine fashion. Reduced synthesis or loss of function through malfolding could adversely affect placental development, for knock-out of the trophoblast specific P0 promoter of *Igf2* in the mouse results in placental and fetal IUGR [47].

## 9 Impact on placental function

The placenta is a major endocrine organ, secreting both peptide and steroid hormones that have a profound effect on maternal physiology and metabolism. The peptide hormones will be processed by the ER, and abnormal glycosylation or folding potentially impacts on their functional capacity. For the syncytiotrophoblast candidate proteins will include hormones such as human chorionic gonadotropin (hCG), placental lactogen (hPL), and placental growth hormone. As mentioned earlier, hCG contains a number of disulfide bonds, whereas hPL is secreted in large quantities, up to 1 g per day, towards term. Both of these hormones are thus vulnerable if normal ER function is perturbed, and so feto-maternal signalling and the capacity of the placenta to influence maternal metabolism may be impaired. This may restrict the supply of glucose and free fatty acids to the placenta.

The syncytiotrophoblast also expresses a wide array of receptors that are involved in signalling and the transport of nutrients. As these are membrane proteins they will be processed by the ER, and so their conformation and activity are potentially compromised during ER stress.

## 10 Placental apoptosis

**Fig. 2:**
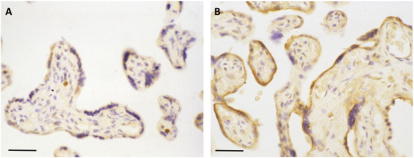
Photomicrograph of placental villi immunolabelled for cleaved caspase 4 from (A) a normal placenta and (B) a case of pre-eclampsia. Both placentas were delivered at term following caesarean section. The normal placenta displays only minimal immunoreactivity, whereas in pre-eclampsia the syncytiotrophoblast covering the villi shows strong staining. Scale bars = 50 μm.

The release of apoptotic debris from the surface of the syncytiotrophoblast is one of the many factors that has been implicated in the second stage of the two-stage model of pre-eclampsia [3]. Microvillous particles and placental debris are highly irritant to endothelial cells *in vitro*, leading to activation and an inflammatory response [48]. Apoptosis is increased in the trophoblast in early-onset pre-eclampsia [49], and ER stress provides at least two potential pathways to mediate this effect, activation of CHOP and of caspase 4. We have observed evidence of both pathways in placentas from early-onset pre-eclampsia, and localised them immunohistochemically to the syncytiotrophoblast and the fetal endothelial cells (Fig. 2). The former may be responsible for increased shedding of placental debris from the syncytiotrophoblast layer, whereas the latter may adversely impact on the development and maintenance of the placental capillary network.

## 11 The association between ER stress and inflammation

A major advance in our understanding of the pathophysiology of pre-eclampsia came with the recognition that the syndrome is associated with a heightened maternal inflammatory response [1,50]. Maternal circulating levels of TNF-α and interleukin 6 are increased in pre-eclampsia [51], and both these cytokines will cause endothelial cell activation. Evidence of such activation is provided by the finding of elevated levels of long pentraxin 3, a marker for inflammation involving a vascular bed, in women with pre-eclampsia [52].

There are close links between ER stress and activation of pro-inflammatory responses that may be mediated by various pathways [53]. Firstly, the kinase domain of Ire1 can activate the p38 MAPK, JNK and NFκB pathways as previously described [54]. Secondly, protein synthesis inhibition independently leads to activation of the NFκB pathway since the half-life of the inhibitory sub-unit, IκB, is much shorter than that of NFκB [55]. Thirdly, the ER produces ROS as a by-product of protein folding, and this may be accentuated during repeated attempts to refold misfolded proteins. ROS can activate the NFκB pathway by stimulating phosphorylation of the IκB sub-unit, targeting it for degradation.

Once activated, these inflammatory responses can form feed-forward loops that build up into vicious cycles, for TNF-α can induce the UPR in a ROS-dependent fashion [10]. The source of the increased TNF-α in the maternal circulation in pre-eclampsia is uncertain, however, although the placenta is an obvious candidate. Oxidative stress *in vitro* and *in vivo* leads to increased tissue concentrations and secretion of the cytokine [7,8,56], and higher concentrations have been reported in pre-eclamptic placentas compared to normal controls [57]. In contrast, a detailed study of non-laboured pre-eclamptic placentas involving sampling from eight independent sites revealed no differences at the mRNA or protein levels compared to controls [58]. These authors concluded that there must be an alternative source of TNF-α, and speculated that this may be activated maternal leucocytes or the endothelium itself.

Despite the widespread recognition that maternal endothelial cell activation represents the second stage of the syndrome, no morphological studies appear to have been performed on peripheral endothelial cells from women with pre-eclampsia. It is therefore impossible to determine at present whether ER stress occurs in these cells, and whether this could contribute to the raised levels of TNF-α. In contrast, there are several reports describing dilation of the ER in the endothelial cells of the umbilical vessels, indicating a loss of ER homeostasis [59,60]. If the same pathology affects the endothelial cells in both circulations during pre-eclampsia, as some authors suspect [61], then it may be that ER stress is not restricted to the placenta in pathological pregnancies. Further investigations are required to explore this possibility.

## 12 Conclusion

Endoplasmic reticulum stress represents one component of a set of integrated cellular responses to stress. There are complex interactions between it and oxidative stress, and it is likely that in many pathologies the two will co-exist. The extensive secretory activity of the syncytiotrophoblast renders it vulnerable to ER stress, and molecular and morphological evidence confirms high levels in placentas from cases of early-onset pre-eclampsia. There will be many consequences for placental development and function, including a reduction in cell proliferation leading to growth restriction, and activation of pro-inflammatory pathways. Potential therapeutic interventions for pre-eclampsia must therefore be designed to address trophoblastic stress in its entirety, rather than individual stress response pathways.
